# Mirusha virus: A novel sand fly-borne phlebovirus with evidence of neutralizing antibodies in humans and dogs in Kosovo

**DOI:** 10.1016/j.onehlt.2026.101488

**Published:** 2026-06-19

**Authors:** Xhevat Jakupi, Elif Kurum, Betim Xhekaj, Ina Hoxha, Julia Walochnik, Vít Dvořák, Donjeta Hajdari, Pranvera Abazi, Adelheid G. Obwaller, Jovana Stefanovska, Aleksandar Cvetkovikj, Kurtesh Sherifi, Remi Charrel, Edwin Kniha, Katharina Platzgummer, Nazli Ayhan

**Affiliations:** a Medical Faculty, University of Prishtina “Hasan Prishtina”, Prishtina, Kosovo; b Unitédes Virus Émergents (UVE: Aix-Marseille Univ, Università di Corsica, IRD 190, Inserm 1207, IRBA), Marseille, France; c Faculty of Agriculture and Veterinary, University of Prishtina, Prishtina, Kosovo; d Center for Pathophysiology, Infectiology and Immunology, Institute of Specific Prophylaxis and Tropical Medicine, Medical University Vienna, Vienna, Austria; e Department of Parasitology, Faculty of Science, Charles University Prague, Prague, Czech Republic; f National Institute of Public Health of Kosovo, Prishtina, Kosovo; g Division of Science, Research and Development, Federal Ministry of Defence, Vienna, Austria; h Department of Parasitology and Parasitic Diseases, Faculty of Veterinary Medicine-Skopje, Ss. Cyril and Methodius University in Skopje, Skopje, Macedonia; i National Reference Center for Arboviruses, Inserm-IRBA, Marseille, France

**Keywords:** Phlebovirus, Sand fly-borne phleboviruses, Republic of Kosovo, Arbovirus, Phenuiviridae

## Abstract

In Europe, phleboviruses are transmitted by phlebotomine sand flies, which are widely distributed in the Mediterranean basin, including the Balkan peninsula. We report the identification and isolation of Mirusha virus (MRSHV), a novel member of the *Phlebovirus corfouense* species within the *Phenuiviridae* family, based on a > 95% genetic identity at the aminoacid level for Large segment between MRSHV and Corfou virus.

MRSHV was isolated from a *Phlebotomus perfiliewi* sand fly specimen collected in Kosovo in 2022. Whole genome sequencing of MRSHV the characteristic trisegmented genome architecture of phleboviruses. Mammalian cells, including green monkey cell lines, were susceptible to MRSHV, with limited replication in dog macrophage cells, whereas no replication was observed in mosquito cell lines, suggesting a vector-specific adaptation. Specific neutralizing antibodies were detected in dogs (2.0%) and in humans (0.8%) from various regions of Kosovo, indicating a broader geographic circulation.

Our findings describe a previously uncharacterized phlebovirus and underscore the importance of considering sand fly–borne phlebovirus infections in cases of undiagnosed febrile illness during the vector-active season, as well as expanding arbovirus surveillance to include hematophagous arthropods beyond mosquitoes.

## 1 Introduction

With recent changes of the International Committee on Taxonomy of Viruses (ICTV), the genus *Phlebovirus* (family *Phenuiviridae)* currently comprises 83 viral species, including three known Old World human pathogenic sand fly-borne viruses [1]; Sandfly fever Naples virus (SFNV; *Phlebovirus napoliense*), Sandfly fever Sicilian virus (SFSV; *Phlebovirus siciliaense*), and Toscana virus (TOSV; *Phlebovirus toscanaense*).

Infections occur related to the seasonal activity of sand flies, typically from April to October. Symptoms of SFSV infections appear after an incubation period of 3 to 5 days, which are high fever, headache, malaise, photophobia, myalgia, and retro-orbital pain. Fevers usually last 2 to 3 days, giving rise to one of the common names for the disease: “three-day fever”, and based on its vectors “sand fly fever” or “pappataci fever”. Leukopenia is often observed during the acute phase of the illness [2]. Although rare, one case from Turkey showed central nervous system infection caused by another Sandfly Fever Virus (SFV) variant, Sandfly Fever Turkey Virus (SFTV) [3].

Notably, SFSV shows a wide geographical distribution including Europe, the Middle East, Central Asia, and Africa. The virus was first isolated from a febrile U.S. soldier in Egypt in 1943 and subsequently identified in Sicily during an outbreak among U.S. troops. Historically, SFSV and SFSV-like virus outbreaks were recorded in countries such as Greece and Serbia, later epidemics and isolated human cases have been documented in Cyprus (Sandfly fever Cyprus virus, SFCV), Turkey (SFTV), and Ethiopia (SFSV-Ethiopia) [4], [5], [6], [7], [8]. In the 1970s, a comprehensive sero-epidemiologic study was performed by Tesh et al. [9] using plaque reduction neutralization tests that revealed the presence of antibodies against SFSV in the human population in a number of countries, including Morocco, Egypt, Turkey, and Portugal [10], [11]. Different seroprevalence studies demonstrating anti-SFSV antibodies in dog and cat sera indicate a circulation of the virus also in Portugal, Greece and Cyprus [12], [13]. SFSV is primarily transmitted by *Phlebotomus papatasi* [14]*.* But there has been increasing direct evidence, with virus isolation or molecular detection, that besides SFSV, there are several closely related but distinct viruses with partly other vector species widely distributed throughout the Mediterranean region and the Middle East: (i) Corfou virus (CFUV; *Phlebovirus corfouense*), discovered in 1981 in sand flies of the *Phlebotomus major* complex on Corfu island in Greece [15]; (ii) CFUV RNA was detected in cerebrospinal fluid (CSF) of a patient in.

Turkey, and this strain was provisionally named Chios-A virus, as CFUV and Chios-A virus sequences showed high genetic similarity [3]; (iii) CFUV was recently detected in sand flies from Bologna, Italy [16]; beside CFUV, Toros virus (TORV; *Phlebovirus torosense*) and Dashli virus (DASHV; *Phlebovirus dashliense*) were isolated from field collected sand flies in Turkey and Iran, respectively [14], [17]. TORV and DASHV are pathogenic for humans and/or animals remains unknown. All of these viruses form a closely related genetic cluster within the SFSV-related phleboviruses, sharing a high degree of sequence similarity and evolutionary relatedness despite their distinct, in some cases, geographical distribution and vector associations.

The known number of different phleboviruses circulating in Balkan countries has recently increased [18], [19], [20]. When historical and recent data are considered together, the Balkan region emerges as a potential hotspot for sand fly–borne viruses, including several of public health relevance such as TOSV and SFSV. The diversity of the phleboviruses appears to be quite high with recent discoveries, and certain areas show the sympatric circulation of multiple phleboviruses [21]. Notably, knowledge of phlebovirus circulation in the Republic of Kosovo remained very limited until recently. Grapi virus (GRPV, *Phlebovirus adanaense*) was discovered in sand flies in the Republic of Kosovo, together with a 13.0% rate of neutralizing antibodies in the local human population and 2.7% in local dogs [19]. On the other hand, seroprevalence studies indicate a high prevalence of SbPV among Austrian soldiers stationed in Kosovo [22]. Additionally, neutralizing antibodies for SFSV have been detected in domestic animals, including sheep, cattle, and dogs in the country [23], [24].

Here, we report the discovery, isolation, and genetic characterization of a novel virus – Mirusha virus (MRSHV), a member of the *Phlebovirus* genus that was identified in sand flies collected in Kosovo. The full genome of MRSHV was sequenced and subjected to phylogenetic analysis. Evidence of human and canine exposure to MRSHV in Kosovo was demonstrated through neutralization assays in serum samples.

## 2 Material and methods

### 2.1 Sand fly trapping and morphological identification

In 2022 and 2023, two independent entomological surveys using CDC miniature light traps (John W. Hock Company, Gainesville, FL, USA) were conducted in all seven regions of the Balkan country the Republic of Kosovo, resulting in 160 sampling sites and 3575 captured sand flies, as published elsewhere [25], [26]. Around 40% of the trapped specimens were morphologically identified. For a time-efficient process to avoid RNA/DNA degradation, of the remaining specimens originating from only five locations, a fraction (minimum of 8%) of the catches per location was identified. Specimens were stored individually or pooled (up to 30 specimens) by sex (ratio 87.2% female vs. 12.8% male), location and feeding status at −80 °C upon homogenization as described in Kurum et al. [19].

### 2.2 Molecular sand fly identification

Molecular confirmation of sand fly species identification from virus-positive samples was performed using PCR-based barcoding of the cytochrome *c* oxidase subunit I (COI) gene [27]. Positive amplicons were purified using the NucleoSpin® Gel and PCR Clean-up Kit (Macherey-Nagel, Germany), followed by Sanger sequencing by Azenta/GENEWIZ (Leipzig, Germany). The resulting sequence data were analyzed using MEGA 12 [28]. The resulting sequences were analyzed by comparison with reference sequences available in the GenBank database.

### 2.3 Processing pools of sand flies and virus detection

Tubes containing individual sand flies or pooled specimens were suspended in Dulbecco's Modified Eagle Medium (DMEM) supplemented with 20% bovine serum albumin, 1% penicillin/streptomycin, 10 μg/mL gentamicin, and 0.25 μg/mL amphotericin B (all reagents from Gibco, Thermo Fisher Scientific). A volume of 500 μL was used for individual flies or pools of up to 15 specimens, while 1000 μL were used for pools containing more than 15 specimens. Each 2.0 mL tube was loaded with two 3 mm metal beads and homogenized using a TissueLyser bead mill (QIAGEN GmbH, Hilden, Germany) at 30 Hz for 1 min. The homogenates were clarified by centrifugation at 14,000 rpm for 5 min at 4 °C.

For RNA extraction, up to 20 individual sand flies were pooled (referred to as super-pool) with using 20 μL of homogenate from each sample and 200 μL of the homogenate from samples with pooled specimens (referred to as pools) were processed using the QIAmp® RNeasy Mini Kit (Qiagen, Hilden, Germany) according to the manufacturer's protocol, with a final elution volume of 50 μL (explained in detail in Kurum et al. 2025) [19].

### 2.4 RT-PCR and virus detection

RT-PCR assays were performed using 5 μL of the extracted nucleic acid. A pan-phlebovirus RT-PCR assay [29] was employed, with the following thermal cycling conditions: reverse transcription at 50 °C for 30 min, initial denaturation at 94 °C for 2 min, followed by 40 cycles of 94 °C for 30 s, 55 °C for 90 s, and 68 °C for 30 s, concluding with a final elongation at 68 °C for 7 min and a hold at 20 °C for 2 min. Positive and negative controls were included in all PCR assays. PCR products were analyzed via electrophoresis on a 2% agarose gel and visualized under UV illumination. Positive products were sequenced using NGS.

The pan-phlebovirus positive samples were subsequently confirmed using a pan-SFSV real-time RT-qPCR assay which can detect CFUV-related viruses as well [13] for which the reagents can be accessed (https://www.european-virus-archive.com/evam-portal-list?portal_search=sicilian) with the 001 K-06231 and − 06236 reference numbers. The primers and positive control for the pan-phlebovirus assay can be accessed at the same address with reference numbers 001 K-06215 and − 06216.

The minimum infection rate (MIR) was calculated based on the assumption that only one individual in each positive pool was infected, using the formula: MIR = (Number of positive pools / Total number of specimens tested) × 1000 [30].

### 2.5 Virus isolation

A 50 μL aliquot of homogenized sand fly material was mixed with 350 μL of enriched Minimum Essential Medium (MEM) supplemented with 1% l-glutamine, non-essential amino acids, 200 mM penicillin-streptomycin, and 3% amphotericin B. This suspension was then inoculated onto Vero E6 cell monolayers. After 1 h of incubation at 37 °C in a 5% CO₂ atmosphere, 2.5 mL of enriched MEM containing 5% fetal bovine serum (FBS) and the aforementioned antibiotics were added to each culture. The cell cultures were observed daily for cytopathic effects (CPE) and subjected to two consecutive passages.

Following each passage, 200 μL of supernatant was collected and screened using standard RT-PCR protocols. Viral stock was generated after the first passage and used for subsequent experiments.

The infectious viral titer was determined using the 50% tissue culture infectious dose (TCID₅₀) assay. Ten-fold serial dilutions of the viral stock were prepared and inoculated onto confluent Vero E6 cells in 96-well plates. TCID₅₀ values were calculated using the Reed and Muench method for estimating the 50% endpoint [31].

### 2.6 Complete genome sequencing and phylogenetic analysis

The phlebovirus PCR positive sample viral stock passage 1 was used for complete genome sequencing for NGS. A total of 200 μL of cell culture supernatant was incubated at 37 °C for 1 h with 30 U of Benzonase (Novagen) and MgCl₂. Viral RNA was extracted using the QIAamp Viral RNA Mini Kit (Qiagen, Hilden, Germany) on a BioRobot EZ1-XL Advanced (Qiagen, Hilden, Germany). Random tagged primers were used for RT-PCR–based random amplification (Applied Biosystems, Waltham, MA, USA). The resulting PCR amplicons were purified using Amicon ultracentrifugal filters (Millipore), and 200 ng of the purified DNA was used for sequencing on the Ion PGM sequencer (Life Technologies SAS, Saint-Aubin, France).

Sequence reads were processed with CLC Genomics Workbench v7.0.4. Reads longer than 30 nt were trimmed with a 99% per-base quality threshold and aligned to reference sequences from Corfou virus strain Pa Ar 814 (GenBank accession numbers KR106177, KR106178, and KR106179 for the L, M, and S segments, respectively). Reads covering at least 50% of the reference sequence and showing a minimum identity of 80% were retained for analysis to balance sensitivity and specificity in the context of genetically diverse field-derived phleboviruses, where stricter cut-offs risk excluding true viral reads. Specific primers were subsequently designed to fill sequencing gaps, and the purified PCR products were re-sequenced by NGS.

The phylogenetic relationships of the novel sand fly-associated phlebovirus MRSHV were investigated by aligning the L, M, and S gene sequences with homologous sequences from selected phleboviruses retrieved from GenBank using ClustalW. Maximum likelihood phylogenetic trees were generated in MEGA12 based on complete amino acid sequences of the RNA-dependent RNA polymerase, glycoproteins N and C, nucleoprotein, and non-structural proteins, applying the LG substitution model. The robustness of the inferred tree topologies was assessed using 1000 bootstrap replicates.

### 2.7 Seroneutralisation test with dog and human sera

Dog serum samples were collected between summer 2021 and spring 2022 as part of a *Leishmania* seroprevalence study in Kosovo, as previously described [32]. Information on age, sex, breed, and health status was recorded for each dog.

Human serum samples were obtained from two laboratories during September–November 2024 and August 2025. Laboratory 1 is a specialized HIV/Hepatitis testing facility, while Laboratory 2 primarily processes TORCH panel requests (Toxoplasma, Rubella, Cytomegalovirus, HSV2), along with some respiratory pathogen tests. Samples from both laboratories were randomly selected to achieve a balanced distribution across geographic regions.

To investigate potential related phlebovirus infection in humans and dogs, we performed seroneutralization assays on 288 dog and 798 human serum samples as previously explained [19]. Heat inactivation was performed on serum samples at 56 °C for 30 min, followed by four serial two-fold dilutions in 96-well microplates. The neutralization step, allowing specific antibodies to bind infectious virus particles and potentially inhibit viral replication, was carried out for 1 h at 37 °C in a final volume of 100 μL containing 100 TCID50 of MRSHV. Subsequently, 100 μL of Vero E6 cell suspension (5 × 10^5^ cells/mL) was added to each serum–virus mixture. Negative and positive controls were included on each plate. All dilution and dispensing procedures, as well as cytopathic effect (CPE) assessment based on the presence or absence of CPE, were automated using the epMotion 5075 system (Eppendorf, Hamburg, Germany) and the Incucyte SX5 Live-Cell Analysis system (Sartorius, Göttingen, Germany) in an NSB3 laboratory. Plates were examined on day 5 post-infection, and samples with titres ≥40 were considered positive for calculation of the overall seroprevalence rate. Viral titres confirmation were determined using the TCID₅₀ assay. Dog serum samples were also tested for Corfou virus (CFUV PaAr 814 strain) to assess cross-reaction.

### 2.8 In vitro viral growth kinetics

Confluent monolayers of Vero African green monkey kidney cells, (Vero-ATCC CCL81), dog (Dh82-CRL-3590 10-year-old male golden retriever macrophage-like) and mosquito (*Aedes albopictus* larvae cells, C6/36, ATCC CRL-1660) cell lines were infected with identified phlebovirus at multiplicities of infection [MOI] of 0.01, 0.1, and 1 in triplicate in 96-well plates. Aliquots of infectious cell culture supernatants were collected every 24 h for an 11-day period. The supernatants were clarified and subsequently extracted using the QIAcube system (Qiagen). Viral genome copies were quantified by real-time RT-PCR [13], employing plasmid-based standards for absolute quantification.

### 2.9 Statistical analysis and mapping of prevalence

Data were prepared with Microsoft Excel for Mac and analyzed with RStudio for Mac (R Core Team 2023). Categorical data [age, breed, region, health status, GRPV seroprevalence, and sex] were analyzed by Fisher's exact test with overall prevalence as the predictor variable. Odds ratios (OR) with exact 95% confidence intervals (CI) were estimated. We refrained from statistical analysis on a municipality level due to the partially low number of available samples. Spearman's rank correlation test was used to assess correlations between neutralization titres (cross-reactions) of identified phlebovirus, TOSV, SFSV, GRPV and CRFV using a two-tailed approach. A two-sided *p*-value <0.05 was considered statistically significant. Prevalence was mapped with QGIS (QGIS Development Team, 2019) using first-level administrative divisions of Kosovo (year 2015) taken from https://earthworks.stanford.edu/catalog/stanford-zh532mm5047.

## 3 Results

### 3.1 Sand fly trapping and morphological identification

Among 3575 sand flies, eight species from two genera were identified: *Phlebotomus perfiliewi*, *Ph. neglectus*, *Ph. tobbi*, *Ph. simici*, *Ph. balcanicus*, *Ph. papatasi*, *Ph. mascittii*, and *Sergentomyia minuta*. *Phlebotomus perfiliewi* and *Ph. neglectus* were most abundant [25], [26].

### 3.2 Molecular sand fly identification

BLAST analysis of the COI sequences from the phlebovirus positive sample showed >99% nt identity with *Ph. perfiliewi* reference sequences in GenBank and clustered together with sequences of recently reported specimens from Kosovo [GenBank accession no. PP296457].

### 3.3 Virus detection and isolation

**Fig. 1:**
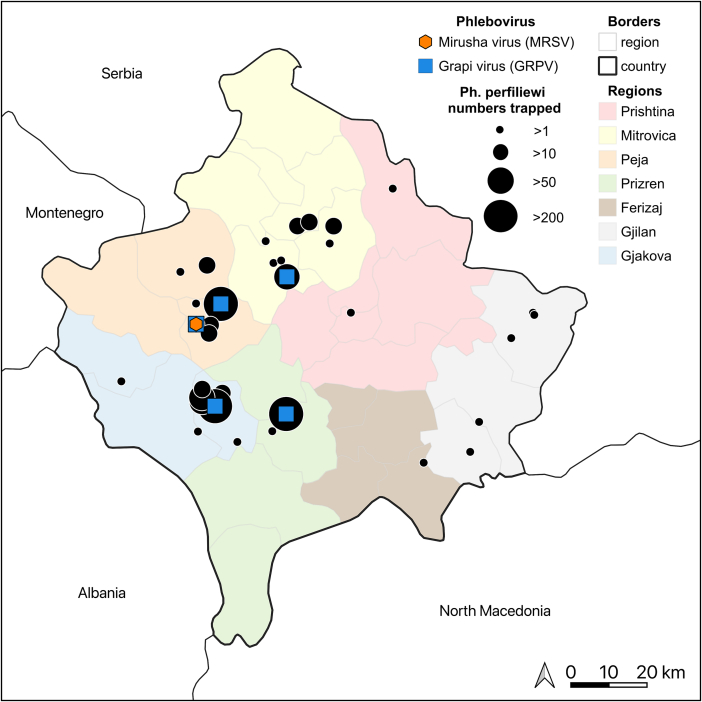
Mapped trapping sites with location positive for Mirusha virus (MRSHV), previous Grapi virus (GRPV) detection [19], and known *Ph. perfiliewi* occurrence shown as absolute trapping numbers [25], [26]. First-level regions of Republic of Kosovo were obtained from https://earthworks.stanford.edu/catalog/stanford-zh532mm5 047 (accessed on 18 July 2023).

All 3575 sand flies were analyzed in 80 pools (containing up to 30 specimens) and 120 super-pools (containing homogenates of up to 20 specimens) [25], [26]. Of these, one super-pool with pooled homogenate of 20 non-engorged female *Ph. perfiliewi* specimens collected in 2022 was positive using the pan-Phlebovirus assay. Subsequent re-testing of all 20 individual specimens using a specific pan-SFSV real-time RT-qPCR assay capable of detecting CFUV-related viruses revealed one positive specimen (3/43), with a Ct value of 29.02, accounting for an MIR of 0.28. Positive and negative controls were included in all PCR assays.The positive sample originated from a location in the region of Peja in western Kosovo (Fig. 1).

Sample #3/43 was inoculated onto Vero E6 cell monolayers and cytopathic effect (CPE) was observed three days post-infection (dpi). The isolate was designated Mirusha virus (MRSHV), named after the Mirusha River located near the collection site of the positive *Ph. perfiliewi* specimen. Following CPE observation, the supernatant was harvested; subsequent analysis via pan SFSV RT-qPCR confirmed active viral replication across successive passages and a viral stock was prepared with a titer of 3.33 × 10^7^ TCID_50_/ml. The passage 1 (P1) isolate of the 3/43 strain was utilized for both seroneutralization assays and viral growth kinetic studies. In addition, the MRSHV 3/43 strain was produced and qualified based on requirements of the European Virus Archive catalog where it is accessible for the scientific community (https://www.european-virus- archive.com) with the following codes: Ref-SKU: 001 V-06374.

### 3.4 Complete genome sequencing and phylogenetic analysis

MRSHV displays the tripartite genome structure characteristic of phleboviruses. Its large segment (L) encodes the RNA-dependent RNA polymerase (RdRp, 6270-nt open reading frame [ORF] [2090 aa]) (GenBank acc no PZ016857). The medium segment (M) encodes a glycoprotein precursor (GPC, 3921-nt ORF [1307 aa]), which is post-translationally cleaved into two viral surface glycoproteins (Gn and Gc) and a non-structural M protein (NSm) (GenBank acc no PZ044334). The small segment (S) encodes a nucleocapsid protein (N) of 741 nt (247 aa) and a non-structural S protein (NSs) of 783 nt (261 aa) (GenBank acc no PZ044335).

The highest pairwise nucleic acid identity between MRSHV and CFUV Pa Ar 814 was 95.36% for the L segment, 90.96% for the M segment and 91.83% for the S segment. According to the ICTV criteria for species delineation which is based on a > 95% genetic identity at the AA level for RdRp [33], MRSHV is a new virus that should be included in the *Phlebovirus corfouense* species.

Protein sequence similarity analysis showed that the MRSHV displays a very high level of similarity to CFUV. The L segment–encoded RdRp; shares 98.71% identity, the M segment–encoded GPC shares 97.63% identity, and the S segment–encoded N and NSs proteins share 100% and 97.39% identity, respectively, with those of CFUV.

**Fig. 2:**
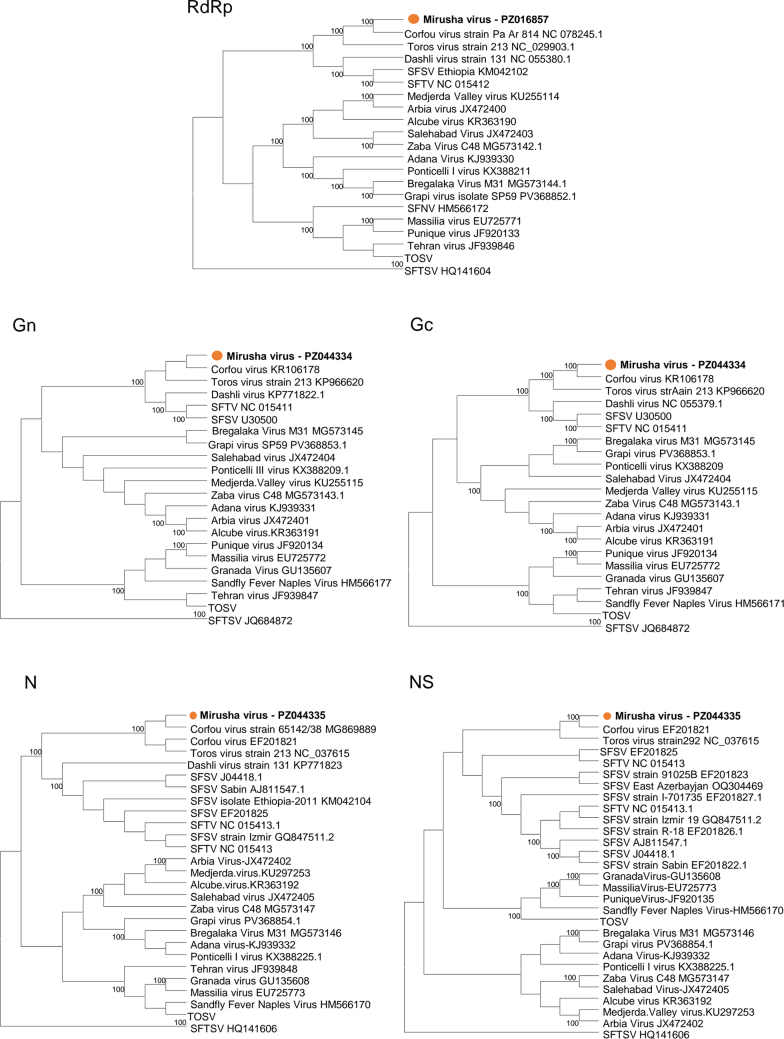
The phylogenetic relationships of phlebovirus amino acid sequences were analyzed based on the L segment RNA-dependent RNA polymerase (RdRp), M segment the glycoprotein N (Gn) and C (Gc) and S segment nucleocapsid (N) and non-structural protein N (Ns).

As expected, phylogenetic analysis showed that the RdRp, Gn, Gc, N, Ns of MRSHV cluster together with CFUV (GenBank accession no. L: KR106177; M: KR106178; S: KR106179) (Fig. 2).

### 3.5 Seroneutralisation of dog and human sera

Of 288 dog sera, 9 (3.1%; 95% CI: 1.5–6.0) showed neutralizing antibodies (NT-Ab) against MRSHV, with neutralization titres ranging from 1:40 (6 sera, 2.0%; 95% CI: 0.9–4.7) to 1:80 (3 sera; 1.0%; 0.3–3.3%). No significant differences in seroprevalence were observed between sexes, health status, breed, or age group, and none of the previously tested *Leishmania*-seropositive dogs had antibodies against MRSHV (Table 1). Seroprevalence was higher in male dogs (4.3%) compared to females (2.0%), and twice as high in mixed breed dogs (4.0%) compared to pure-bred (1.8%). A significant increased association was observed between MRSHV-positivity and anti-SFSV antibodies (OR = 7.4, *P*-value = 0.04), whereas no significant differences were shown for TOSV and GRPV (Table 1).

**Table 1:** Canine MRSHV seroprevalence associated with various risk factors. TOSV (Toscana virus), SFSV (Sandfly fever Sicilian virus), GRPV (Grapi virus), CFUV (Corfou virus).

Parameter (sample size)	Positive (%)	OR (95% CI), P-value
Sex		
Female (147)	3 (2.0%)	reference
Male (141)	6 (4.3%)	2.1 (0.4–13.4), 0.21
Health status		
Normal (246)	8 (3.3%)	reference
Disrupted (42)	1 (2.4%)	0.7 (0.02–5.7), 1
Breed		
Mixed (177)	7 (4.0%)	reference
Pure-bred (111)	2 (1.8%)	0.5 (0.05–2.4), 0.75
Age group		
0–4 (165)	7 (4.2%)	reference
4–8 (91)	1 (1.1%)	0.3 (0.01–2.0), 0.5
>8 (32)	1 (3.1%)	0.7 (0.02–6.0), 1
Leishmania IFAT^a^		
Negative (275)	9 (3.3%)	–
Positive (13)	0 (0.0%)	–
TOSV^b^		
Negative (252)	8 (3.2%)	reference
Positive (36)	1 (2.8%)	0.9 (0.02–6.8), 1
SFSV^b^		
Negative (277)	7 (2.5%)	reference
Positive (11)	2 (18.2%)	7.4 (0.8–53.9), *0.04*
GRPV^b^		
Negative (280)	9 (3.2%)	–
Positive (8)	–	–
CFUV^c^		
Negative (284)	5 (1.8%)	–
Positive (4)	4 (100%)	–

a Previously tested in Xhekaj et al. [32].

b Previously tested in Kurum et al. [19].

c Tested for cross-reactivity with MRSHV.

Of 798 human sera tested, 6 (0.8%, 95% CI: 0.3–1.7) showed neutralizing antibodies (NT-Ab) against MRSHV, with neutralization titres of 1:40 (4 sera, 0.5%; 95% CI: 0.2–1.4), up to 1:160 (2 sera, 0.3%; 95% CI: 0.04–1.0). No significant difference was observed between women and men as well as between age groups (Table 2). Increased MRSHV seropositivity of 2.6% (OR = 3.4, not significant) was observed for humans previously tested for anti-GRPV antibodies (Table 2).

**Table 2:** Human MRSHV seroprevalence associated with sex, age group, and Grapi virus (GRPV) seropositivity..

Parameter (sample size)	Positive (%)	OR (95% CI), *P*-value	
Sex			
Female (546)	4 (0.7%)	reference	
Male (252)	2 (0.8%)	1.1 (0.1–7.6), 1	
Age group			
1–10 years (35)	–	–	
11–20 years (44)	–	–	
21–30 years (199)	2 (1.0%)	reference	
31–40 years (171)	2 (1.2%)	1.2 (0.1–16.2), 1	
41–50 years (98)	1 (1.0%)	1.0 (0.01–19.7), 1	
51–60 years (97)	1 (1.0%)	1.0 (0.02–19.9), 1	
61–70 years (109)	–	–	
>70 years (45)	–	–	
GRPV^a^			
negative (520)	4 (0.8%)	reference	
positive (78)	2 (2.6%)	3.4 (0.3–24.1), 0.18	

a Only 598 sera tested for GRPV in a previous study.

Spearman's rank correlations were used to assess cross-reactions in dog sera between TOSV, SFSV, GRPV, CFUV, and a significant correlation was observed only between CFUV, and MRSHV (0.7, P-value≥ 0.001) which highlight that these two viruses are in the same viral species, while no significant correlations were found between all other phleboviruses, suggesting a distinct immunological response with no evidence of cross-reactivity (Supplementary Table 1).

### 3.6 Seroprevalence by region

**Fig. 3:**
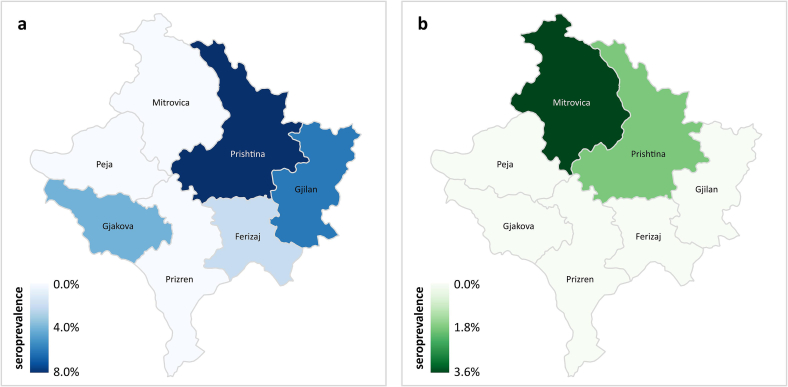
Canine (a) and human (b) MRSHV seroprevalence by region.

The seroprevalence of MRSHV in dog sera ranged from 0.0% in Mitrovica, Peja, and Prizren, 2.4% (95% CI: 0.1–14.4) in Ferizaj, 2.6% (95% CI: 0.1–15.1) in Gjakova, to 7.5% (95% CI: 2.0–22.5) and 8.0% (95% CI: 2.6–20.1) in Gjilan and Prishtina region, respectively (Fig. 3a).

In humans, MRSHV seroprevalence varied from 0.0% in Peja, Prizren, Ferizaj, Gjilan, and Gjakova to 0.7% (95% CI: 0.2–1.9) in Prishtina and 3.6% (95% CI: 0.6–13.6) in Mitrovica region (Fig. 3b).

Due to the small number of samples in some municipalities, we refrained from conducting a detailed analysis and present MRSHV seroprevalence by municipality in Supplementary Fig. 1 and Supplementary Table 1.

### 3.7 In vitro viral growth kinetics

**Fig. 4:**
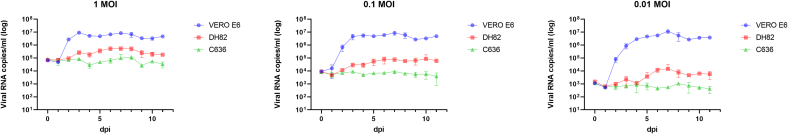
In vitro replication kinetics of Mirusha virus (MRSHV). Growth curves were generated in African green monkey kidney (Vero E6; orange), canine macrophage (DH82; yellow), and *Aedes albopictus* (C6/36; green) cell lines at multiplicities of infection (MOI) of 1, 0.1, and 0.01. Supernatants were harvested at 24 h intervals up to 12 days post-infection (dpi). Viral load (genomic RNA copies/mL) was quantified via RT-qPCR. (Limit of detection (LoD) of the real-time RT-PCR assay is 2,22 copy per μl). (For interpretation of the references to colour in this figure legend, the reader is referred to the web version of this article.)

The in vitro growth kinetics of MRSHV in Vero E6 cells demonstrated a rapid increase in viral load starting at 2 days post-infection (dpi). Cultures reached a plateau at 4 dpi for multiplicities of infection (MOI) of 1 and 0.1, while the 0.01 MOI peaked at 8 dpi. Maximum genomic titers on Vero E6 cells ranged between 10^6 and 10^7 RNA copies/mL. Conversely, MRSHV replication in DH82 canine macrophage cells was significantly more protracted. For all the MOIs' viral titers increased by a maximum of one log. In contrast, C6/36 mosquito cells were entirely non-permissive, with no detectable viral replication observed throughout the 12-day observation period (Fig. 4).

## 4 Discussion

The diversity of phleboviruses in the Balkans is likely driven by a combination of ecological, climatic, and landscape factors that favour both sand fly abundance and virus maintenance. The region lies at the intersection of Mediterranean, continental and sub-Mediterranean climate zones, creating a mosaic of temperature, humidity, and precipitation regimes that supports high sand fly species richness and extended seasonal activity. Complex topography, including coastal areas, river valleys, and fragmented rural landscapes, further generates diverse microhabitats and breeding sites for *Phlebotomus* vectors. In addition, land-use patterns such as traditional agriculture, animal husbandry, and peri-domestic habitats increase contact between vectors, wildlife, and domestic hosts, facilitating enzootic and possibly spillover transmission cycles. Climate variability and warming trends may also be expanding the suitable ecological niche for key vector species, thereby enhancing viral persistence and geographic spread [21], [34].

MRSHV represents the second sand fly-borne phlebovirus identified and isolated in Kosovo, following the discovery of GRPV [19]. The taxonomic divergence between these two viruses underscores a significant diversity of phleboviruses and highlights their active co-circulation within the region. Geographically positioned at the crossroads of Europe and Asia, Kosovo's humid continental climate provides an excellent niche for sand fly vectors, and shows a decreasing southwestern to northeastern winter temperature trend, which is also reflected by species richness and the heterogenic modelled climatic suitability for sand flies in the country [35]. Specifically, *Ph. perfiliewi* and *Ph. neglectus* have been identified as the predominant species supporting viral maintenance [25].

The detection of MRSHV in *Ph. perfiliewi* from the Peja region in western Kosovo is consistent with the well-documented high density of this species in the area [25]. Given that several phleboviruses, such as TOSV and GRPV, have been previously isolated from *Ph. Perfiliewi* [25], [35]*,* these findings reinforce the role of this species as a versatile vector capable of harboring and potentially transmitting multiple phlebovirus lineages.

Based on a > 95% genetic identity at the AA level for RdRp between MRSHV and CFUV, which was previously the unique member of the *Phlebovirus corfouense* species, MRSHV is classified as a novel member of the species. This finding extends the known geographic range of the species beyond Greece and Italy to include Kosovo. The historical identification of CFUV in 1981, combined with its detection in Italy in 2016 and our current findings, suggests a stable, decade long presence of this viral lineage within the Mediterranean and Balkan regions. Furthermore, while CFUV was originally associated with *Phlebotomus major* in Greece, its presence in Italy and the isolation of MRSHV from *Ph. perfiliewi* in Kosovo suggest a broader vector range than previously assumed, as suggested also for TOSV [36]. Future research should combine longitudinal field surveillance with experimental infection assays in colonized sand flies. Such studies are necessary to define the biological and environmental drivers that determine the transmission efficiency of different phleboviruses.

In contrast to GRPV, which was detected across four distinct regions in Kosovo (Fig. 1) [19], MRSHV was identified in only a single specimen (voucher #3/43). This localized detection raises questions regarding inter-viral competition, particularly given that both viruses share the same vector species. Noteworthy, MRSHV was detected in sand flies originating from one of the GRPV-positive sites, indicating localized co-circulation [19]. However, it remains to be determined whether the dominance of GRPV suggests competitive exclusion or if specific ecological constraints limit the broader distribution of MRSHV within these sympatric populations. However, the lack of long-term surveillance of phleboviruses in Kosovo and insufficient number of samples tested highlight the need of further investigation for confirmation of this claim.

The MRSHV infection rate in sand flies was 0.28% which is lower than other phleboviruses identified in sand flies such as Zaba virus (0.41%), Adria virus (0.45%) and Alcube virus (0.45%) and higher than those observed for Adana virus (0.01%) and Medjerda Valley virus (0.02%) [18]. The low MIR of 0.28 in sand flies coincides with the modest seroprevalence observed in dogs (2.0%) and humans (0.8%). These figures are notably lower than those reported for GRPV, which exhibits seropositivity rates of 2.7% in dogs and a significantly higher rate of 13.0% in humans [19]. The uniform distribution of MRSHV antibodies across canine demographics – regardless of sex, age, breed, or health status – suggests that exposure is likely ubiquitous and driven by environmental factors rather than host-specific traits. Furthermore, the lack of antibody overlap in *Leishmania*-seropositive dogs confirms a lack of cross-reactivity between these pathogens. Similar patterns in humans further support the notion that environmental or ecological factors, rather than individual host traits, primarily determine the risk of MRSHV infection. However, seroprevalence rates and titers are low, MRSHV-positive human sera were detected in two different regions, while positive dog sera were identified in four regions, with only one overlapping region. In addition, the virus-positive sand fly was detected in the Peja region. Altogether, MRSHV-positive findings were observed in six of the seven regions of Kosovo, supporting a broad geographic distribution of the virus within the country. Although, the low seropositivity rates observed across the study area limits the identification of a distinct regional pattern, the presence of neutralizing antibodies directly indicates an infectious process that elicits an immune response in both dogs and human and may indicate the low circulation of the virus. Notably, despite the initial isolation of MRSHV in the Peja region, both human and canine samples from this region tested negative for neutralizing antibodies. This discrepancy may be attributed to various factors that need further attention. Firstly, trapping numbers of *Ph. perfiliewi* varied considerably across regions, which influences the probability of viral detection as observed in our study. However, the cross-sectional set-up of these studies does not allow inference on actual abundance [25], [26]. Secondly, the isolation of MRSHV from a single *Ph. perfiliewi* specimen does not rule out other sand fly species as potential vectors, such as *Ph. neglectus*, which is most widely present in the county [25], [26]. Specifically in north-eastern parts, where human and canine MRSHV seroprevalence was observed. Consequently, further large-scale longitudinal studies are required to clarify the correlation between vector abundance and local seroprevalence.

A significant association was observed between MRSHV seropositivity and the presence of anti-SFSV antibodies in dogs, a trend not observed for TOSV or GRPV. This correlation is probably due to the close phylogenetic and antigenic relationship between SFSV and MRSHV. As members of a shared evolutionary lineage, these viruses likely possess conserved epitopes, potentially leading to serological cross-reactivity in neutralization or binding assays. These results raise the possibility of detecting seroneutralisation antibodies against closely related phleboviruses such as SFSV and since the positive samples against both SFSV and MRSHV are limited, this result should therefore be interpreted with caution.

To evaluate viral tropism and host cell permissivity, we conducted in vitro growth kinetic analyses using non-human primate (VeroE6), canine (Dh82) and mosquito (C6/36) cell lines. Our results demonstrated high susceptibility to MRSHV in Vero E6 cells, which yielded peak genomic titers as observed with other sand fly-borne phleboviruses (Fig. 2). Notably, C6/36 mosquito cells were non-permissive to MRSHV infection. This lack of replication is significant given that C6/36 cells are typically highly susceptible to a broad range of arboviruses due to a dysfunctional antiviral RNA interference (RNAi) response [37]. These findings indicate that the MRSHV viral tropism aligns with that of SFSV [38]. Furthermore, dog macrophage cells exhibited low permissivity, suggesting that dogs may experience transient, low-level viremia rather than high-titer systemic infection, might indicate potentially acting as accidental or ‘dead-end’ hosts in the MRSHV transmission cycle. This could be as well due to the fact that Dh82 are immune competent and may not support higher replication compared to Vero E6 which are immuno (IFN)-defficient. Therefore, this in-vitro growth kinetics data may not inform the complicated and dynamic virus replication within the host.

MRSHV detection in vectors, together with its genetic and in vitro characterization and the presence of neutralizing antibodies in both humans and dogs against the virus, indicates active circulation of the virus in nature and underscores its relevance within a One Health framework.

Chios-A virus, a virus closely related to CFUV, was detected in the CSF of a patient in south of Turkey with viral encephalitis and no travel history, suggesting potential central nervous system infections of the virus [3].

Although there have been no formally confirmed human cases of CFUV infections, including MRSHV, beyond this observation, the close genetic relationship with Chios-A virus, as well as with other known pathogenic phleboviruses such as SFSV, SFCV, and SFTV, which cause mainly febrile illness signs and symptoms, suggests a possible medical relevance [2]. The substantial burden of febrile illness outbreaks associated with SFSV variants in Turkey, Greece, Egypt and Ethiopia [6], [7], [8], [39] further supports the hypothesis that MRSHV and related viruses may contribute to undiagnosed febrile and neurological infections in humans and possibly also animals in the Balkan region.

Several limitations of this study should be acknowledged. First, the in vitro growth kinetics experiments were performed with using only bimolecular technics in Vero E6, DH82 canine macrophage, and mosquito-derived C6/36 cell lines, without evaluation in human cell lines. In addition, DH82 cells are immune-competent and may inherently restrict viral replication compared with Vero E6 cells, which are interferon-deficient. Therefore, the observed replication dynamics may not fully reflect the complex interactions and viral kinetics occurring in vivo within natural hosts. The absence of animal infection models further limits conclusions regarding MRSHV pathogenicity, tissue tropism, duration of viremia, and transmission dynamics. Another limitation concerns the specificity of the seroneutralization assays. The close antigenic relationship among related phleboviruses, particularly SFSV-like viruses, may affect the interpretation of serological findings. Finally, the human serum sampling strategy may not accurately represent the general population, as samples were obtained from individuals seeking HIV/hepatitis testing or TORCH screening, populations that may differ in demographic structure, health status, and exposure risk. Consequently, the observed seroprevalence could represent either an underestimation or an overestimation of the true prevalence of MRSHV exposure in the general population of Kosovo.

Given this context, a broad RT-qPCR assay targeting all SFSV and SFS-like viruses, is avaible not only to detect MRSHV but also to identify close relatives or novel viruses within this pathogenic phlebovirus group [13]. Indeed, MRSHV belongs to a large cluster of phleboviruses in which human pathogens are more frequent than in other phlebovirus lineages, underscoring the importance of continued surveillance and diagnostic efforts.

## 5 Conclusion

To fully assess the public health implications of MRSHV, future research should concentrate on: (i) longitudinal surveillance to define seasonal transmission patterns; (ii) in vivo pathogenicity studies to determine virulence and clinical relevance; (iii) detailed entomological mapping to clarify the ecological niche of its vectors; and (iv) prospective clinical cohort studies to evaluate the role of MRSHV in unexplained febrile illnesses. Altogether, the characterization of MRSHV reinforces the view that the Balkan Peninsula represents a critical biogeographical hotspot for the emergence and diversification of phleboviruses.

Overall, these findings highlight the need to strengthen One Health surveillance of sand fly-borne viruses in the Balkans, alongside continued entomological, ecological, virological, and clinical studies to clarify the role of MRSHV and related phleboviruses in human and animal health.

## CRediT authorship contribution statement

**Xhevat Jakupi:** Writing – original draft, Methodology, Investigation, Conceptualization. **Elif Kurum:** Writing – original draft, Methodology, Investigation, Data curation. **Betim Xhekaj:** Writing – review & editing, Methodology, Investigation, Data curation. **Ina Hoxha:** Writing – review & editing, Project administration, Conceptualization. **Julia Walochnik:** Writing – review & editing, Supervision, Conceptualization. **Vít Dvořák:** Writing – review & editing, Project administration, Methodology, Conceptualization. **Donjeta Hajdari:** Writing – review & editing, Investigation. **Pranvera Abazi:** Writing – review & editing, Investigation, Data curation. **Adelheid G. Obwaller:** Writing – review & editing, Methodology, Conceptualization. **Jovana Stefanovska:** Writing – review & editing, Methodology, Conceptualization. **Aleksandar Cvetkovikj:** Writing – review & editing, Methodology, Conceptualization. **Kurtesh Sherifi:** Writing – review & editing, Methodology, Data curation, Conceptualization. **Remi Charrel:** Writing – review & editing, Supervision, Data curation, Conceptualization. **Edwin Kniha:** Writing – original draft, Supervision, Project administration, Data curation, Conceptualization. **Katharina Platzgummer:** Writing – original draft, Supervision, Data curation, Conceptualization. **Nazli Ayhan:** Writing – review & editing, Writing – original draft, Project administration, Methodology, Formal analysis, Data curation, Conceptualization.

## Ethical approval

Dog serum samples were tested as remnants of a previously published study on Leishmania seroprevalence (Xhekaj et al. 2023), which was conducted in compliance with the regulations of the Department of Hygiene, Welfare, and Ethology of Animals, Faculty of Agriculture and Veterinary, University of Prishtina Hasan Prishtina. Sampling was performed following the approval of the faculty on 19 March 2021. Scientific research works that include investigation of vector-borne emerging diseases in dogs are performed to diagnose animal diseases and improve animal welfare. No suffering was caused during the sample collection.

Human sera were tested based on prior anonymous unlinked collection from the laboratories conducting serological testing at the Department of Microbiology, National Institute of Public Health (NIPH), following Ethical Approval from the Institutional Review Board of the NIPH Kosovo on 12 June 2025 and of the Kosovo Chamber of Doctors on 9 July 2025 with the protocol number of 214/2025.

## Funding

The study has been funded by the Austrian defense research program FORTE of the Federal Ministry of Finance (BMF) (Project number: 886318) and partially funded by the Ministry of Education, Science, Technology and Innovation of the Republic of Kosovo (Project number: 2-1775). The funders had no role in the study design, data collection and analysis, decision to publish, or preparation of the manuscript. Elif Kurum is financially supported by the Study Abroad Postgraduate Education Scholarship (MEB1416), awarded by the Republic of Türkiye Ministry of National Education.

## Declaration of competing interest

The authors declare the following financial interests/personal relationships which may be considered as potential competing interests: Nazli Ayhan and other authors, declare that they have no known competing financial interests or personal relationships that could have appeared to influence the work reported in this paper.

## Data Availability

Data will be made available on request.

## Appendix A Supplementary data

Supplementary Table 1. Correlation between canine seropositivity of various phleboviruses. TOSV=Toscana virus, SFSV=Sandfly fever Sicilian virus, GRPV=Grapi virus, CRFV=Corfou virus. Correlation index (bottom left, color scale), P-value (top right, italics grey).

Supplementary Figure 1. Canine (a) and human (b) MRSHV seroprevalence by municipality.

Supplementary Table 2. Canine and human seroprevalence by municipality. Missing data indicated by NA.
