# Supplementary material for: Mirusha virus: A novel sand fly-borne phlebovirus with evidence of neutralizing antibodies in humans and dogs in Kosovo
Source: One Health. 2026 Jun 19;23:101488. doi: 10.1016/j.onehlt.2026.101488 (PMC13320489; doi:10.1016/j.onehlt.2026.101488)
Supplement: Supplementary file 1 — Supplementary Table 1. Correlation between canine seropositivity of various phleboviruses. TOSV=Toscana virus, SFSV=Sandfly fever Sicilian virus, GRPV=Grapi virus, CRFV=Corfou virus. Correlation index (bottom left, color scale), P-value (top right, italics grey). [file mmc1.docx]

**Supplementary Table 1.** Correlation between canine seropositivity of various phleboviruses. TOSV=Toscana virus, SFSV=Sandfly fever Sicilian virus, GRPV=Grapi virus, CFUV=Corfou virus, Mirusha virus=MRSHV. Correlation index (bottom left, color scale), *P*-value (top right, italics grey).

| **Phlebovirus** | **TOSV** | **SFSV** | **GRPV** | **CFUV** | **MRSHV** |
| --- | --- | --- | --- | --- | --- |
| **TOSV** | - | *0.56* | *1* | *0.4* | *0.9* |
| **SFSV** | 0.03 | - | *0.57* | *0.65* | *<0.01* |
| **GRPV** | 0 | -0.03 | - | *0.7* | *0.6* |
| **CFUV** | -0.05 | -0.02 | -0.02 | - | *<0.001* |
| **MRSHV** | -0.08 | 0.17 | -0.03 | 0.7 | - |
