# Supplementary figures and images for: Mirusha virus: A novel sand fly-borne phlebovirus with evidence of neutralizing antibodies in humans and dogs in Kosovo
Source: One Health. 2026 Jun 19;23:101488. doi: 10.1016/j.onehlt.2026.101488 (PMC13320489; doi:10.1016/j.onehlt.2026.101488)

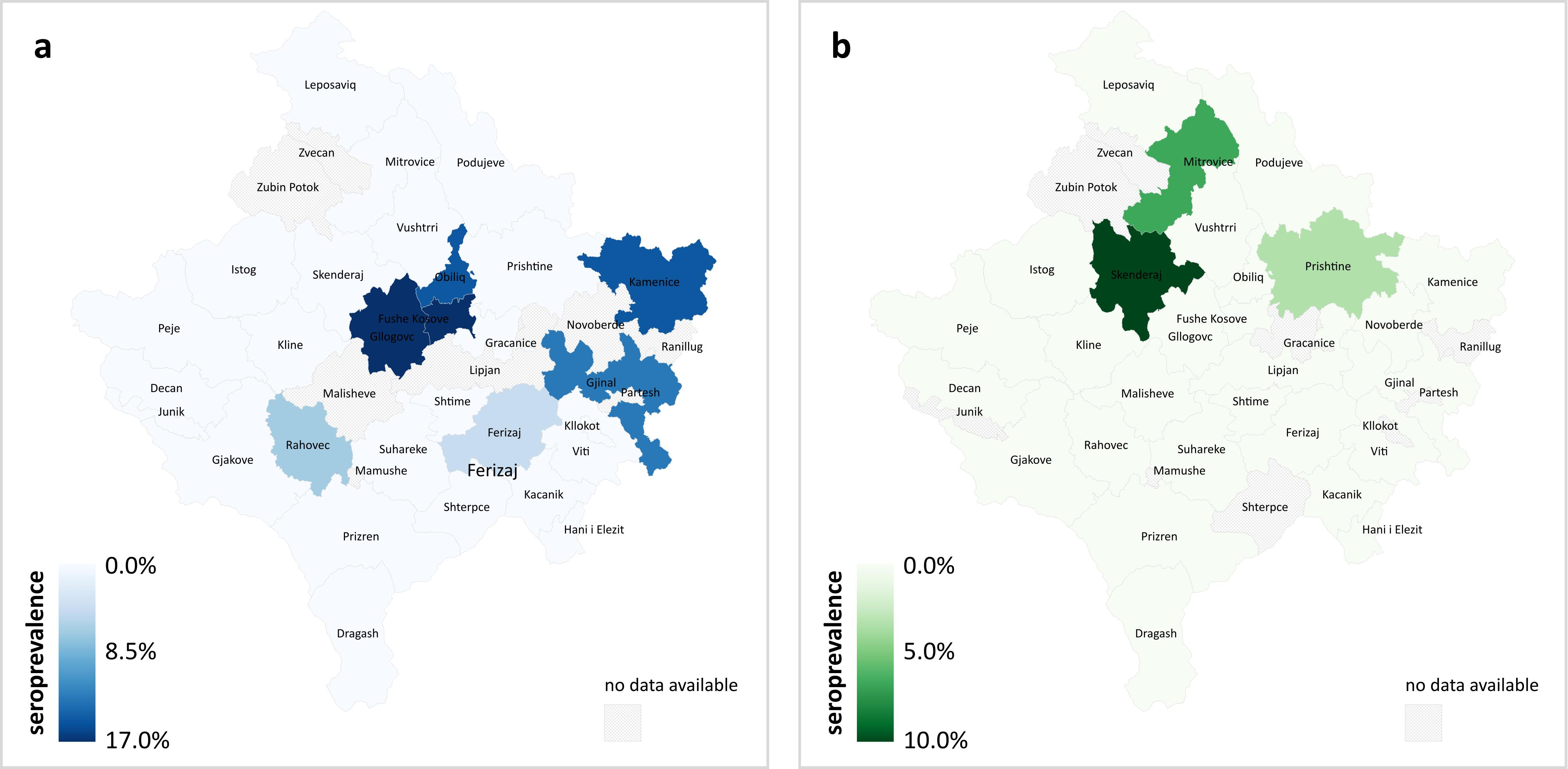

Supplement: Supplementary file 2 — Supplementary Figure 1. Canine (a) and human (b) MRSHV seroprevalence by municipality. [file mmc2.zip › mmc2_EK.png]
