# Supplementary material for: Mirusha virus: A novel sand fly-borne phlebovirus with evidence of neutralizing antibodies in humans and dogs in Kosovo
Source: One Health. 2026 Jun 19;23:101488. doi: 10.1016/j.onehlt.2026.101488 (PMC13320489; doi:10.1016/j.onehlt.2026.101488)
Supplement: Supplementary file 3 — Supplementary Table 2. Canine and human seroprevalence by municipality. Missing data indicated by NA. [file mmc3.docx]

**Supplementary Table 2.** Canine and human seroprevalence by municipality. Missing data indicated by NA.

| **municipality** | **canine – positive/total (%)** | **human –positive/total (%)** |
| --- | --- | --- |
| Decan | 0/2 (0.0%) | 0/3 (0.0%) |
| Dragash | 0/8 (0.0%) | 0/1 (0.0%) |
| Ferizaj | 1/19 (5.3%) | 0/40 (0.0%) |
| Fushe Kosove | 1/6 (16.7%) | 0/49 (0.0%) |
| Gjakove | 0/13 (0.0%) | 0/7 (0.0%) |
| Gjinal | 1/8 (12.5%) | 0/15 (0.0%) |
| Gllogovc | 1/6 (16.7%) | 0/18 (0.0%) |
| Gracanice | 0/8 (0.0%) | NA |
| Hani i Elezit | 0/6 (0.0%) | 0/3 (0.0%) |
| Istog | 0/12 (0.0%) | 0/4 (0.0%) |
| Junik | 0/7 (0.0%) | NA |
| Kacanik | 0/6 (0.0%) | 0/2 (0.0%) |
| Kamenice | 2/14 (14.3%) | 0/3 (0.0%) |
| Kamenice-Petroc | NA | NA |
| Kline | 0/6 (0.0%) | 0/4 (0.0%) |
| Kllokot | 0/2 (0.0%) | NA |
| Leposaviq | 0/2 (0.0%) | 0/1 (0.0%) |
| Lipjan | NA | 0/34 (0.0%) |
| Malisheve | NA | 0/18 (0.0%) |
| Mamushe | NA | NA |
| Mitrovice | 0/13 (0.0%) | 1/18 (5.6%) |
| Novoberde | NA | 0/1 (0.0%) |
| Obiliq | 2/14 (14.3%) | 0/30 (0.0%) |
| Partesh | NA | NA |
| Peje | 0/20 (0.0%) | 0/20 (0.0%) |
| Podujeve | 0/6 (0.0%) | 0/29 (0.0%) |
| Prishtine | 0/10 (0.0%) | 4/401 (1.0%) |
| Prishtine-Shashkoc | NA | NA |
| Prizren | 0/19 (0.0%) | 0/31 (0.0%) |
| Rahovec | 1/17 (5.9%) | 0/2 (0.0%) |
| Ranillug | NA | NA |
| Shterpce | 0/5 (0.0%) | NA |
| Shtime | 0/5 (0.0%) | 0/13 (0.0%) |
| Skenderaj | 0/13 (0.0%) | 1/10 (10.0%) |
| Suhareke | 0/13 (0.0%) | 0/8 (0.0%) |
| Viti | 0/16 (0.0%) | 0/6 (0.0%) |
| Vushtrri | 0/12 (0.0%) | 0/27 (0.0%) |
| Zubin Potok | NA | NA |
| Zvecan | NA | NA |
